# Association between health literacy and dietary intake of sugar, fat and salt: a systematic review

**DOI:** 10.1017/S1368980020002311

**Published:** 2021-06

**Authors:** Alessandra Buja, Giulia Grotto, Laura Montecchio, Elisa De Battisti, Milena Sperotto, Chiara Bertoncello, Silvia Cocchio, Tatjana Baldovin, Vincenzo Baldo

**Affiliations:** Department of Cardiological, Thoracic and Vascular Sciences, and Public Health, University of Padova, Padova 35127, Italy

**Keywords:** Prevention, Chronic disease, Sugar, Salt, Fat, Health literacy

## Abstract

**Objectives:**

To collect and summarise all current data from observational studies, generating evidence of the association between health literacy (HL) and the dietary intake of sugar, salt and fat, to analyse intervention studies on the promotion of an appropriate dietary intake of the above-mentioned nutrients and to ascertain whether HL moderates the efficacy of such intervention.

**Design:**

A systematic literature search of analytical observational studies on the association between HL and dietary intake of sugar, salt and fat was performed in Medline and Scopus databases. Intervention studies on the promotion of healthy nutrition that concerned the intake of sugar, salt and fat were also assessed.

**Results:**

Of the eight observational studies included in this review, five investigated dietary intake of sugar, one focused on salt, one assessed sugar and salt and one analysed the fat intake. The results of the five studies assessing sugar were mixed: three found an association between low levels of HL and a high sugar intake, one found this association only for boys and two found no evidence of any association. The two studies assessing salt and the one assessing fat found no evidence of any association with HL. One intervention study on the sugar intake concluded that HL was not a significant moderator of the intervention’s effectiveness.

**Conclusion:**

No evidence of any association between HL and salt and fat intake emerged, while for sugar, the results are mixed. More work is needed to better understand the moderating effects of HL on the outcomes of health promotion interventions.

In recent years, it has become overwhelmingly clear that regularly eating foods that are high in energy, such as saturated fats, with added sugar and salt, is among the key risk factors for developing weight problems, metabolic disorders and related diseases^(1–3)^. In this regard, the WHO has produced specific recommendations on the appropriate dietary intake of the above-mentioned elements to prevent the rise of non-communicable diseases^(4)^. In particular, total fat should not exceed 30 % of the total energy intake, saturated fat should be <10 % of the total energy intake and *trans*-fat <1 %. Fat consumption should shift away from saturated fats and *trans*-fats to unsaturated fats and towards the goal of eliminating industrially produced *trans*-fats. As regards the intake of free sugars, this should be limited to <10 % of the total energy intake, and <5 % is suggested for additional health benefits. Keeping salt intake to <5 g a day (corresponding to Na intake of <2 g a day) helps to prevent hypertension and reduces the risk of heart disease and stroke in the adult population^(4)^.

By the mid-1900s, this trio of ingredients – salt, sugar and fat – had taken on a new psychosensory dimension because the processed food industry had discovered that they could be formulated to generate a sense of satiety and pleasure in consumers^(5)^. Palatable food activates brain reward circuitry through fast sensory inputs and slow post-ingestive consequences (such as raising glucose concentration in the blood and brain). The repeated supraphysiological stimulation of reward pathways may make people’s behaviour increasingly compulsive and lead to further loss of control over food intake and ultimately to obesity^(6,7)^. It is well known that the foods that are more palatable and that induce more cravings are typically processed foods or foods with a high salt, sugar and/or fat content^(5)^. Today, crave-inducing foods and soft drinks are sold and consumed worldwide, creating an unfavourable food environment that has led to higher rates of obesity and its co-morbidities, diabetes and CVD^(8,9)^.

From the above considerations, there is an abundantly clear need to implement all useful strategies to induce people to lower their consumption of sugar, salt and fat in an effort to improve the population’s general health and prevent the non-communicable diseases that are currently the main causes of death. Providing the population with appropriate dietary information and promoting a healthy eating behaviour are certainly essential to efforts in this sense.

One factor that has gained importance in recent years, as evidence has emerged of its association with health-related behaviours (such as diet), is health literacy (HL)^(10)^, or an individual’s ability to access and understand health-related information and consequently make appropriate health-related decisions. HL empowers individuals, enabling them to take responsibility for their health^(11,12)^. A limited HL has an adverse impact on health-related behaviour and disease-preventing strategies, such as participation in screening programmes^(13–16)^. In addition, people with a limited HL tend to make more use of healthcare services, including hospitalisations and emergency services, partly as a consequence of their often adopting unhealthy lifestyles^(17–19)^. HL can thus play a fundamental part in health promotion and prevention strategies^(20)^, and its importance was emphasised at the Ninth Global Conference on Health Promotion (Shanghai, 2016)^(21)^. Moreover, the theoretical model of health promotion indicated that improving HL is a way to improve people’s control and thereby change intermediate health outcomes such as dietary habits. However, no syntheses of empirical evidence have yet been provided. Concerning nutrition and dietary behaviour, it was recognised that higher levels of literacy, numeracy and HL are predictors of food label use, good portion-size estimation skills, lower BMI and better dietary quality^(22–25)^. However, there are still several aspects that remain to be clarified, for example, if HL affects the intake of specific nutrients.

The first aim of this systematic review was to shed more light on the link between HL and dietary intake of sugar, salt and fat. The second aim was to discover the role of baseline HL levels in influencing the effectiveness of health promotion programmes aiming to encourage appropriate sugar, salt and fat intake. To achieve the first aim, we collected and summarised all currently available data emerging from observational studies generating evidence of the association between HL and dietary intake of sugar, salt and fat. To assess the second aim, we considered intervention studies promoting a healthy diet that referred specifically to an appropriate sugar, salt and fat intake, and we analysed their outcomes to see whether HL moderated the efficacy of such intervention.

## Methods

### Search strategy

For the current study, a comprehensive and systematic literature search was conducted in the Medline and Scopus databases to identify:

1. observational studies (cross-sectional, cohort and case–control studies) investigating the association between HL and dietary intake of sugar, fat and salt;
2. intervention studies aiming to promote an appropriate intake of these three ingredients to see whether HL influences the efficacy of the intervention.

The search process involved the use of three different search strings obtained by combining the terms ‘health literacy’ or ‘literacy’ with the terms ‘sugar*’ or ‘sweet*’ or ‘sport drink’, and ‘salt*’ or ‘salty’ or ‘sodium’, and ‘fat*’ or ‘fatty’ using Boolean operators. Three different search strings were used (one for each of the nutrients considered), so the search yielded three different lists of papers. The search strings were defined as explained in online supplementary material, Appendix 1.

The records retrieved from the databases were imported in Endnote and duplicates were removed. Two reviewers (G.G. and L.M.) checked the search hits by reading the article titles and abstracts. If the results of a study were published more than once, only the most complete article was considered in the analysis. The authors also checked the reference lists of the papers included in the review for any articles not already considered.

### Data extraction

The following data were extracted from each study: first author’s name, year of publication, journal, study design, sampling method, characteristics of the study sample (e.g., age range), measures of outcome and exposure, results, confounding factors, interactions and the author’s conclusions.

### Eligibility criteria

The studies included in the review had to meet the following inclusion criteria: HL had to be measured using validated questionnaires; dietary intake of sugar, fat and salt had to be measured quantitatively; a measure of the association between HL and dietary sugar/fat/salt intake had to be reported and the paper had to have been published from January 2000 up until January 2019 (for sugar) or March 2019 (for salt and fat) and written in English. Studies involving patients with a specific disease and previous systematic reviews were ignored. For the intervention studies, only explicit interventions aimed at reducing salt, sugar or fat intake were considered in this systematic review.

### Quality assessment criteria

Two different authors (G.G. and L.M.) independently judged the methodological quality of the studies using the Strengthening the Reporting of Observational Studies in Epidemiology approach^(21)^ for observational studies and the Consolidated Standards of Reporting Trials approach^(22)^ for randomised controlled trials. Total Strengthening the Reporting of Observational Studies in Epidemiology and Consolidated Standards of Reporting Trials scores were calculated for each study. A larger percentage of items conforming to the guidelines indicated a higher methodological quality: ≥80 % excellent quality, 60–79 % good quality, 50–59 % sufficient quality and <50 % poor quality. A Cohen’s kappa score was calculated to establish the level of agreement between the two reviewers’ assessments.

## Results

The reference lists of the nine selected articles yielded no additional papers meeting our inclusion and exclusion criteria. The review was thus conducted on nine papers in all (one paper assessed the association between HL and both sugar and salt intake). Among these, eight were observational studies, while one was a randomised controlled trial.

Considering these nine articles, the number of participants enroled in each observational study ranged from 100 to 3165. Seven studies^(23–29)^ were conducted on adults (one of them^(23)^ only enroled participants over 60 years old), while one study sample^(30)^ consisted of dyads involving a female caregiver and a 3- to 5-year-old child, and one study was conducted on adolescents aged 13–15 years^(31)^.

The studies were conducted in various parts of the world, including the USA (five studies), Iran (two studies), Switzerland (one study) and Indonesia (one study), and they were published between 2012 and 2018.

HL was measured with the Newest Vital Sign tool in three studies (one of them considered only three of the six items in the test) and with the Rapid Estimate of Adult Literacy in Medicine – Short Form^(32)^ in two studies. Other tools used in single studies were the Rapid Estimate of Adult Literacy in Dentistry-30^(33)^, the Nutrition Literacy Questionnaire (NLQ-20)^(34)^, the Oral Health Literacy – Adults Questionnaire^(35)^ and the Test of Functional Health Literacy in Adults – Shortened version. Two tests were administered in one study: a Short Food Literacy Questionnaire for adults^(36)^ and the sixteen items concerning the health promotion domain in the European Health Literacy questionnaire (HLS-EU-Q47, German version)^(37)^.

The studies varied in their approach to measuring the outcomes and relied mostly on self-reported information. In the majority of cases, participants answered questionnaires about their nutritional habits; only one study estimated Na intake (in g/d) from urinary excretion. Tables 1 and 2 provide details of the observational studies identified and included in the review.

**Table 1:** Overview of studies reviewed. Material and methods

Author Year Journal	Children (C) Adult (A)	Study design Sample methods	Sample Age	Measure of exposure	Measure of outcome
Sugar					
Divaris 2012 Acta Odontol Scand	Child–caregiver dyads	Cross-sectional study Data were taken from the Carolina Oral Health Literacy project. Purposeful quota sampling was used to ensure adequate study sample representation of minority groups	North Carolina, USA Two hundred three female caregiver/child dyads Inclusion criteria: caregivers with children aged 3–5 years who completed the Early Childhood Oral Health Impact Scale Exclusion criteria: males, Asians and first language other than English	OHL of parents measured with Rapid Estimate of Adult Literacy in Dentistry-30 Scores 0–30, with low OHL < 13	Consumption of sweets (never/occasionally, once a day, more than once a day)
Irwan 2016 International Journal of Nursing Sciences	A	Cross-sectional study Participants were selected from a list of older adults drawn up by a nurse responsible for monthly health check-ups and a community leader in the district of Tammua	Indonesia 2014 One hundred forty older adults (over 60 years) Inclusion criteria: age over 60 years, ability to hear and see, no mental disorders and independence in activities of daily living	REALM-SF: seven-item word recognition test consisting of health-related terms Scores from 0 (= third-grade reading level or below might be unable to read most material) to 7 (HL level considered = high school education)	Having sugar and sugary food/beverage limitations (never, seldom, sometimes, always)
Joulaei 2018 Progress in Nutrition	C	Cross-sectional study Adolescents aged 13–15 years were selected from secondary schools using a cluster random sampling method	Shiraz, Iran Three hundred eighty-eight adolescents Inclusion criteria: age between 13 and 15 years, attending school in Shiraz Exclusion criteria: foreign students and adolescents with chronic diseases or special diets	Nutrition Literacy Questionnaire (NLQ-20): this comprises thirty-four items measured on a five-point Likert scale from 1 (strongly agree) to 5 (strongly disagree) including the three main domains of nutrition literacy NLQ-20 scores ranged from 20 to 100	Added sugar consumption in % of total energy intake, measured using the Revised Children’s Diet Quality Index, and assessed with a validated FFQ
Persoskie 2017 Prev Chronic Dis	A	Cross-sectional study Data from the Health Information National Trends Survey Round 4 Cycle 3 (mailed questionnaire). Households were randomly selected using a stratified sample from a listing of all non-vacant residential addresses in the USA. One adult per household was asked to complete the questionnaire	USA Year 2013 Three thousand one hundred sixty-five participants Inclusion criteria: age 18+ years, resident in USA	NVS Score range 0–4 The study excluded two questions from the NVS (Would it be safe to eat the ice cream if you are allergic to penicillin, peanuts, latex gloves and bee stings? If not, why not?) due to space limitations on the study instrument	Dietary behaviours: the number of days respondents drank sugar-sweetened soda or pop each week (every day; 5–6 d a week; 3–4 d a week; 1–2 d a week; <1 d a week; I do not normally drink any soda or pop)
Sistani 2017 Eur J Dent	A	Cross-sectional population-based survey Multistage random area served as the sampling method, and the sample size was calculated by estimating a single proportion	Tehran, Iran 2011 One thousand thirty-one adults Inclusion criteria: 18–65 years old Exclusion criteria: illiteracy in Persian	OHLAQ Seventeen items in four sections: reading comprehension, numeracy, listening and decision-making. Correct answers = 1; incorrect or missing answers = 0. OHLAQ scores are divided into three levels: inadequate (0–9), borderline (10–11) and adequate (12–17)	Consumption of sugary snacks or beverages between daily meals, categorised as ‘once or more daily (≥1/d)’ and ‘less than once daily (<1/d)’ as an optimal oral health behaviour.
Zoellner 2011 J Am Diet Assoc.	A	Cross-sectional survey Participants were recruited by seven indigenous Delta residents (community health workers and/or Extension Programme Assistants) A proportional quota sampling plan based on educational achievement levels was developed and used to assure that participants were representative of the greater Delta region. Recruiters were also trained to recruit across all age ranges (>18 years of age), an equal number of men and women, and approximately 70 % African American and 30 % white participants	Arkansas and Louisiana, USA Three hundred seventy-six participants Inclusion criteria: age 18+ years, resident in the rural Lower Mississippi Delta, English speaking	NVS Scores range 0–6 High likelihood of limited literacy 0–1 score Possibility of limited literacy 2–3 score Adequate literacy skills 4–6 score	Dietary intake: validated 158-item FFQ to construct HEI scores. Score range 0–100 The sum of twelve component scores including total fruit (including 100 % fruit juice) (5 points), whole fruit (5 points), total vegetables (5 points), dark green and orange vegetables and legumes (5 points), total grains (5 points), whole grains (5 points), milk (10 points), meat and beans (10 points), oils (10 points), saturated fat (10 points), Na (10 points) and energy content from solid fat, alcoholic beverages and added sugars (20 points). Higher scores indicate better adherence to DGA recommendations SSB consumption: summing kilocalories from five items on the FFQ, including carbonated soft drinks, fruit drinks, powdered drink mixes, coffee with sugar and tea with sugar
Zoellner 2016 Int J Behav Nutr Phys Act	A	Randomised controlled trial Recruitment strategies: active (e.g., recruitment at health departments) and passive (e.g., flyers, newspaper ads, word of mouth). Randomisation at individual level. Participants engage in one of the two types of intervention (MoveMore or SipSmarter)	Southwest Virginia, USA Years 2012–2014 Three hundred one low socio-economic status adults from medically underserved rural regions were enroled and randomised (155 allocated to SipSmarter; 146 allocated to MoveMore) Two hundred ninety-six participants completed the programme Inclusion criteria: English-speaking adults ≥18 years of age, with self-reported consumption of ≥200 SSB kcals/d, no self-reported contraindications for physical activity, regular access to a telephone and not concurrently enroled in another nutrition or physical activity programme. To minimise cross-contamination between intervention groups, only one member per household was allowed to enrol in the trial	NVS. Six-item questionnaire to assess HL based on the nutrition facts panel. Scores 0–3: high likelihood or possibility of limited literacy; scores 4–6: adequate HL	BEVQ-15, a validated food frequency instrument that assesses the past month’s beverage consumption Theory of Planned Behaviour questions related to SSB consumption (twenty items) and physical activity (twenty items)
Salt					
Irwan 2016 International Journal of Nursing Sciences	A	Cross-sectional study Participants were selected from a list of older adults prepared by a nurse responsible for MHC and a community leader in the district of Tammua	Indonesia 2014 One hundred forty older adults (over 60 years) Inclusion criteria: age over 60 years, ability to hear and see, no mental disorders and independence in activities of daily living	REALM-SF: seven-item word recognition test consisting of health-related terms Scores from 0 (= third-grade reading level or below might be unable to read most material) to 7 (HL level considered = high school education)	Internal self-care practices were measured with the Health Promoting Lifestyle Profile II questionnaire; out of fifty-two questions, six were related to salt consumption (e.g., ‘I limit use of salt and salty foods’). Response options were ‘never’, ‘seldom’, ‘sometimes’ or ‘always’.
Luta 2018 Nutr Metab Cardiovasc Dis	A	Cross-sectional study Participants were employees of eight social services, production/service, university/research or public service organisations in the German-speaking part of Switzerland, all with staff canteens The sample size of 145 employees exceeded a threshold of 112 participants, calculated to be necessary to detect a reduction in the primary end point (24-h urinary Na excretion) by 15 % with *α* 0·05 and 80 % power, which would also allow for a 10 % drop-out rate	Switzerland April–October 2015 One hundred forty-five participants, 15–65 years old Inclusion criteria: use of staff canteen at least once a week, availability every 3 months for nutrition workshops held during regular working hours and sufficient knowledge of German Exclusion criteria: medical or nonmedical issues and/or use of medication that would influence urine collection and analysis	HL was measured selectively using the sixteen items in the health promotion domain of the validated European Health Literacy questionnaire (HLS-EU-Q47, German version). HL index (0–50 points): inadequate (≤25), problematic (>25–33), sufficient (>33–42) or excellent (>42–50) Food literacy was measured using a validated, twelve-item questionnaire scored using four- or five-point Likert scales and covering crucial elements of nutrition literacy and FL definitions. A summary score was calculated that ranged from 7 to 52; the higher the score, the better the FL	Prediction of Na and K intake from urinary excretion (g/d)
Fat					
Guntzviller 2017 J Immigr Minor Health	A	Cross-sectional study Two bilingual university extension employees who routinely work with low-income, Spanish-speaking populations helped identify and recruit participants	Lake County, Indiana, USA One hundred adults, Spanish-speaking with limited or no English proficiency and 200 % below the poverty line	Short version of the Spanish Test of Functional Health Literacy in Adults. Two short exercises from the reading comprehension and two from the numeracy sections were used to calculate the scale. HL scores ranged from 1 to 100, with scores above 66 corresponding to an ‘adequate’ HL.	Answer to the question ‘Do you consistently avoid eating high-fat foods?’ Answer sets included: No, I do not intend to in the next 6 months (1), No, but I intend to in the next 6 months (2), No, but I intend to in the next 30 d (3), Yes, I have been, but for <6 months (4), Yes, I have been for more than 6 months (5)

OHL, oral health literacy; REALM-SF, Rapid Estimate of Adult Literacy in Medicine, Short Form; HL, health literacy; NVS, Newest Vital Sign; OHLAQ, Oral Health Literacy − Adults Questionnaire; SSB, sugar-sweetened beverage; FL, food literacy; HEI, Healthy Eating Index; DGA, Dietary Guidelines for Americans; BEVQ, Beverage Intake Questionnaire; MHC, monthly health checkups.

**Table 2:** Overview of studies reviewed. Results

Author Year Journal	Results	Confounders	Conclusion	Additional comments (e.g., details of intervention)
Sugar				
Divaris 2012 Acta Odontol Scand	Although a positive trend emerged, the frequency of the children’s sweet consumption was not found significantly associated with the caregivers’ level of oral HL (only a bivariate distribution was performed).	Age Education level Race	OHL was not significantly associated with child’s oral health-related quality of life	
Irwan 2016 International Journal of Nursing Sciences	No association was found between HL level and sugar limitation	Gender Occupation Education level Health status	Sugar limitation correlated with salt limitation (0·15, *P* < 0·05) and MHC – Monthly Health Check-ups (0·10)	
Joulaei 2018 Progress in Nutrition	Among boys, an increasing FNL (component of total nutrition literacy) was associated with higher sugar score quartile, which showed lower intake of sugar (OR 1·071, 95 % CI 1·002, 1·146, *P* < 0·05)	Gender BMI Parents’ education level Physical activity	A higher FNL was associated with lower sugar intake and a better energy balance in boys	
Persoskie 2017 Prev Chronic Dis	In unadjusted models, NFP label understanding was negatively associated with sugar-sweetened soda consumption: participants with a better understanding of the NFP reported consuming sugar-sweetened soda fewer days a week (OR 0·88; 95 % CI 0·81, 0·94; *P* = 0·001). After adjusting for demographic characteristics, the association between NFP label understanding and sugar-sweetened soda consumption remained significant (OR 0·90; 95 % CI 0·81, 0·99; *P* = 0·03)	Age Gender Education level Income Race/ethnicity	Many consumers have difficulty interpreting nutrition labels and label understanding correlates with self-reported dietary behaviours (those with better label understanding tended to drink less sugar-sweetened soda) Higher scores for label understanding were associated with consuming less sugar-sweetened soda, even after adjusting for demographic factors	
Sistani 2017 Eur J Dent	High OHL scores 12–17 correlated significantly with a lower consumption of sugary snacks or beverages (<1/d between meals) (OR = 1·56, 95 % CI 1·13, 2·15, *P* = 0·007)	Age Gender Education level Economic status	Age and OHL scores predict individuals’ consumption of sugary drinks and snacks (<1/day between meals). This result indicates that all adults, and especially youngsters and those with limited literacy skills, should have access to easily understood messages about a diet for oral health.	
Zoellner 2011 J Am Diet Assoc.	HL significantly predicted SSB beverage consumption (*R* ^2^ = 0·15; *F* = 6·3; *P* < 0·01), while accounting for demographic variables. For SSB, participants in the lowest HL category consumed about 119 kcal/d more than those with an adequate HL Every 1 point in HL scores was associated with 34 fewer SSB kcal/d	Age Gender Education level Household income Race SNAP participation	An important relationship exists between HL and SSB consumption and illustrates how understanding the causes and consequences of limited HL is an important factor in promoting compliance with the Dietary Guidelines for Americans	
Zoellner 2016 Int J Behav Nutr Phys Act	HL status did not significantly influence 6-month retention rates (low HL = 79 ± 41 %, high HL = 72 ± 45 %; *P* = 0·56) or class engagement rates (low HL = 2·14 ± 1·03, high HL = 1·97 ± 1·15; *P* = 0·20) The 6-month SSB kcal reduction between participants in the SipSmarter condition with low HL *v*. high HL was not statistically significant (*P* = 0·21).	Age Gender Education level Income Race/ethnicity Employment status Number of children Smoking status BMI HL	Baseline HL status did not moderate any of the primary or secondary outcomes. Between the low HL and high HL groups, the relative effect of the treatment was not statistically significant (*P* = 0·31).	SipSmarter targeted decreasing SSB consumption, with the primary goal of achieving the SSB recommendation of <8 fluid ounces a day. To sufficiently target SSB reduction, participants were educated on recommendations for all beverage categories (including water, unsweetened beverages, milk). The comparison condition, MoveMore targeted PA promotion, with the primary goal of achieving 150 min of moderate-intensity aerobic activity and doing muscle-strengthening activities on two or more days a week. The final 6-month intervention structure, informed by the preliminary work, included three small-group classes, one live teach-back call and eleven Interactive Voice Response calls. SipSmarter and MoveMore conditions were matched in duration and contact. Each of the small-group classes was 90–120 min in duration and delivered in weeks 1, 6 and 17
Salt				
Irwan 2016 International Journal of Nursing Sciences	No association was found between HL level and salt limitation	Gender Occupation Education level Health status	The number of respondents who never limited their sugar and salt intake was especially surprising. An intervention programme should be developed to limit salt and sugar intake by the Indonesian elderly	
Luta 2018 Nutr Metab Cardiovasc Dis	In multiple regression analysis, neither HL nor FL was significantly associated with salt intake	Age Gender	There was no significant association between the HL index or FL scores and salt intake. The only variable significantly associated with salt intake (*P* = 0·005) was how salt content impacts food/menu choice. The analysis strongly emphasises the need to raise health-related knowledge, abilities and skills regarding Na/salt and K in the working population of Switzerland and, in particular, to bridge the gap between knowledge and salt-related dietary practices	
Fat				
Guntzviller 2017 J Immigr Minor Health	HL and avoiding fatty foods were not significantly associated	Age Gender Race Education level	Self-efficacy interacted with HL in avoiding fatty foods although the interaction was only marginally significant. As participants’ HL increased, the positive relationship between self-efficacy and avoiding fatty foods became stronger	

HL, health literacy; OHL, oral health literacy; FNL, functional nutrition literacy; NFP, Nutrition Facts panel; SSB, sugar-sweetened beverage; FL, food literacy; SNAP, Supplemental Nutrition Assistance Program.

### Health literacy and sugar intake

**Fig. 1:**
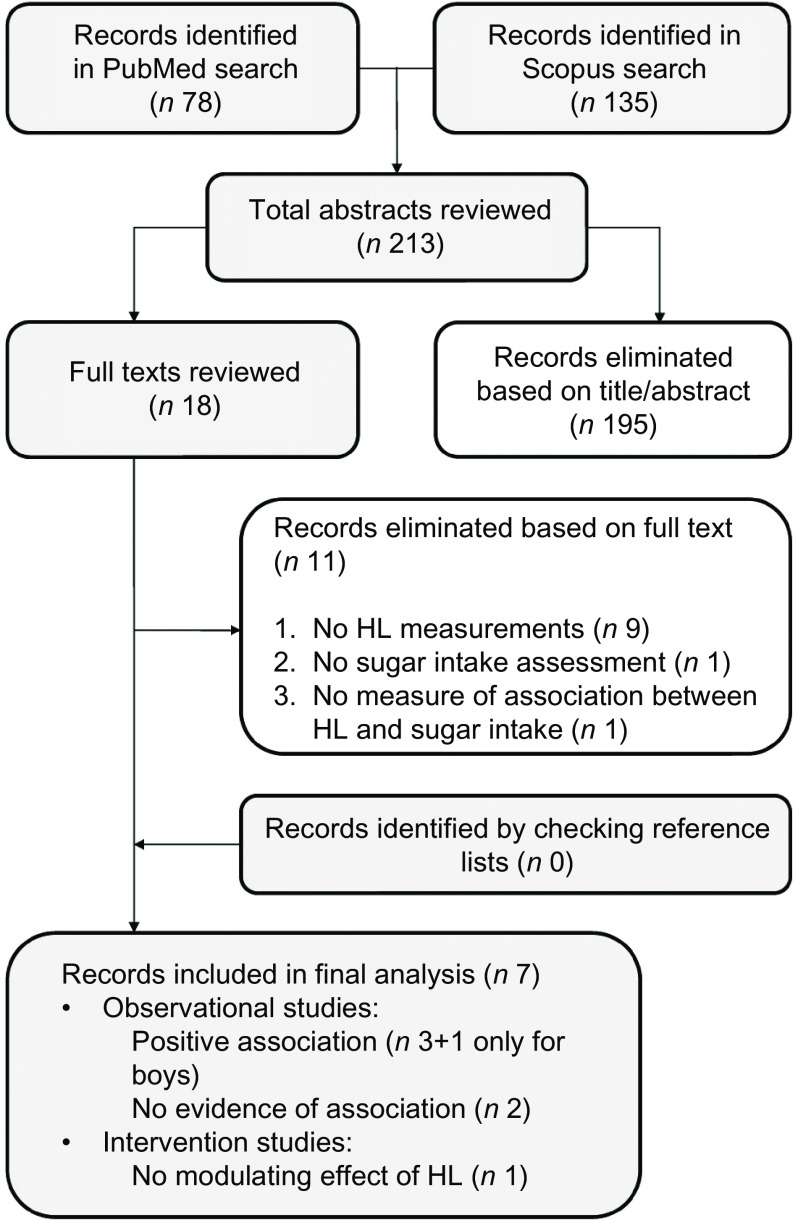
Article selection process (sugar), flow chart. HL, health literacy

Figure 1 shows the flow chart of the article selection process for the association between sugar intake and HL. Seven articles met our inclusion criteria and were included in this review: three studies found that participants with a higher HL had a lower sugar intake, one found the same association only for boys and two found no evidence of any association.

All three studies finding an association were conducted on adults. Two of these studies were conducted in the USA and focused specifically on sugar-sweetened beverage (SSB) consumption, using the Newest Vital Sign tool to measure participants’ HL. Using nationally representative data, Huizinga *et al.* ^(24)^ found that participants with a better understanding of panels reporting nutritional facts reported consuming sugar-sweetened soda fewer times a week. Their findings were consistent with those of von Elm *et al.* ^(26)^, who reported that participants in the lowest HL category (as measured with the Newest Vital Sign tool, which measures understanding of food labels) ingested just over 100 kcal/d more from SSB than participants with an adequate HL. Each additional point in participants’ HL scores (0–6) was associated with a 34 kcal/d lower intake from SSB. The third study showing an association between a higher HL and a more limited consumption of sugar was conducted by Cha *et al.* ^(25)^, who investigated the association between oral health-related behaviour and oral HL among Iranian adults. They considered the consumption of sugary snacks or beverages between meals, finding that higher oral HL scores correlated significantly with a lower consumption of these foods (less than once a day).

Oral HL was assessed by Naghibi Sistani *et al.* too^(30)^, who investigated the association between caregivers’ oral HL and their children’s oral health-related quality of life. The authors explored whether HL modified the association between children’s oral health status and oral health-related quality of life. The study was based on data from structured interviews with female caregivers of 3- to 5-year-old children to examine deleterious oral health practices in children. Although a trend emerged, the frequency of the children’s sweet consumption was not found significantly associated with the caregivers’ level of oral HL (only bivariate distribution was performed). The only study that we found concerning adolescents (13–15 years old) estimated their eating behaviour using a validated FFQ and examined its association with nutritional literacy^(31)^. This construct was investigated specifically in three aspects – functional nutrition literacy, interactive nutrition literacy and critical nutrition literacy – using the Nutrition Literacy Questionnaire (NLQ-20)^(34)^. It emerged that a higher functional nutrition literacy was associated with a lower sugar intake in boys, while there was no evidence of such an association in girls. Sugar intake was not associated with either of the other aspects of nutritional literacy (interactive nutrition literacy or critical nutrition literacy) in either sex.

A single cross-sectional study aimed to examine self-care practices and health-seeking behaviours of older adults (over 60 years)^(23)^. Various self-care practices were investigated, including the dietary intake of sugar and salt. No evidence of any association emerged between HL and a limited consumption of these elements.

The only intervention study we found was a randomised and controlled trial aiming to examine the effects of a behavioural intervention to reduce SSB intake among adults in medically underserved rural communities^(27)^. It emerged that baseline HL status did not moderate any outcomes.

### Health literacy and salt intake

**Fig. 2:**
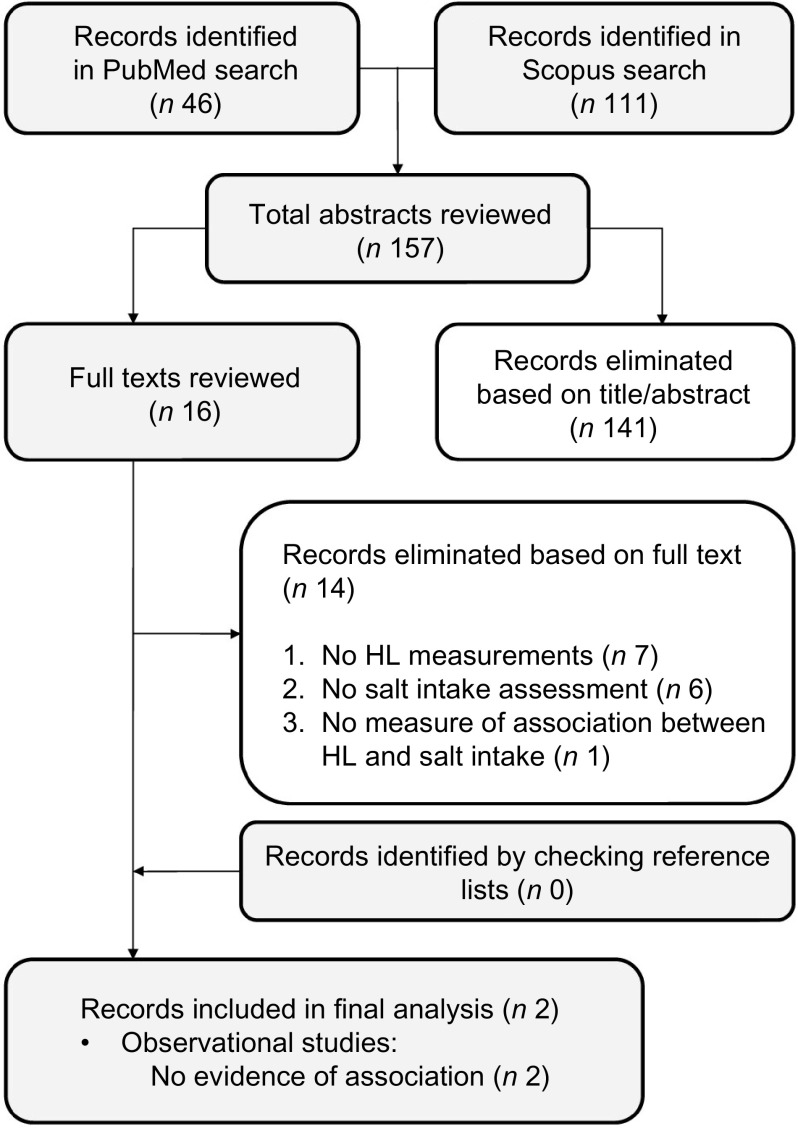
Article selection process (salt), flow chart. HL, health literacy

Figure 2 shows the flow chart of the article selection process for the association between salt intake and HL. Two cross-sectional studies were selected. Neither found any significant association between salt intake and HL. Irwan *et al.* ^(28)^ sought associations between HL, food literacy (FL) and salt awareness, on the one hand, and salt intake, K intake and Na:K ratio, on the other hand, using baseline data from a workplace intervention trial conducted in Switzerland. Na and K intakes were estimated from a single 24-h urine collection. The authors found a tendency towards a lower salt intake among the more health-literate and food-literate participants, but on multiple regression analysis, neither HL nor FL was significantly associated with salt intake, K intake or Na:K ratio. The only variable significantly associated with salt intake was food salt awareness when it came to the choice of foods.

### Health literacy and fat intake

**Fig. 3:**
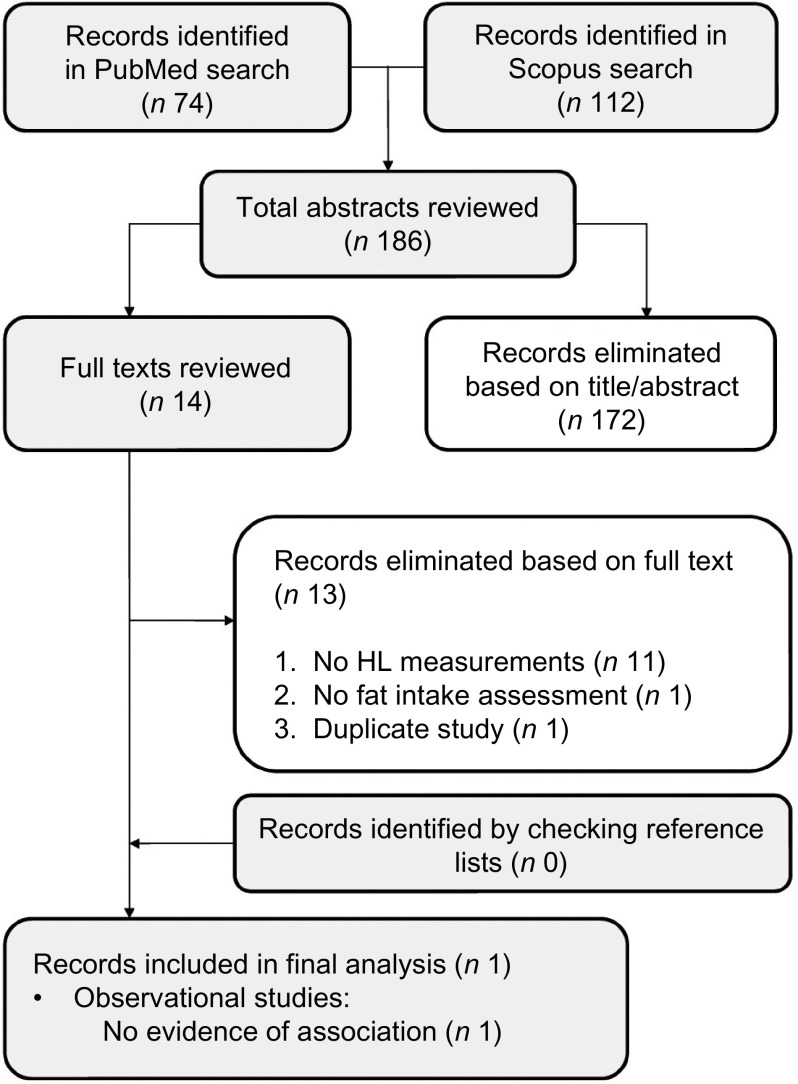
Article selection process (fat), flow chart. HL, health literacy

Figure 3 shows the flow chart of the article selection process for the association between fat intake and HL. The only study investigating this issue aimed to examine HL, self-efficacy and eating and exercising behaviour in a low-income Hispanic population in the USA^(29)^. The results produced no evidence of any statistically significant association between HL and the intake of fatty foods. Self-efficacy interacted with HL as regards to the three types of behaviour investigated (eating fruits and vegetables, avoiding fatty foods and exercising), but the interaction was only marginally significant for avoiding fatty foods.

Appendix 2 in the online supplementary material outlines the methodological quality of the studies. Overall, of the eight observational studies considered, three were of good, three of moderate and two of poor methodological quality. The single randomised controlled trial was compliant for 77 % of the items. A Cohen’s kappa of 0·66 reflected a good agreement between the two reviewers.

## Discussion

Three of the six observational studies that considered sugar intake found that a greater HL coincided with a lower sugar intake, providing weak evidence of HL being a determinant of sugar intake, mainly in adults. As for salt and fat intake, our review goes to show that the evidence to support any empirical associations between HL and salt and fat is still very scanty, and no studies have found an association between HL and these nutrients to date.

Returning to the association between HL and sugar intake, the three studies revealing an association between a greater HL and a lower sugar intake (especially in the form of SSB) were conducted on adults. These findings indicate that healthier dietary practices are associated with a better understanding of food labels and suggest that HL might have a role in improving adult people’s diet. Given that SSB consumption has doubled over the past three decades in the USA^(38–40)^, the relationship between these drinks and obesity^(41–43)^, and the evidence of their consumption being inversely related to the level of schooling^(44)^, and to HL, would warrant major targeted intervention to reverse the trends of their consumption^(26)^. This consideration would not apply to elderly people, as the only study on this age group found no association between HL and sugar intake.

While children and young adults are a core target group for HL research and practice, there are limited knowledge and academic consensus regarding the abilities and knowledge a child or young person should possess in order to make sound health-related decisions. The only study that investigated the link between nutritional literacy and eating behaviour in adolescents found that a higher nutritional literacy is associated with a lower sugar intake in boys, but not in girls^(36)^.

Only one randomised and controlled trial examined the effects of a behavioural intervention to reduce SSB intake and see if HL influenced retention, engagement or outcomes^(27)^. It emerged that baseline HL status did not moderate any of the primary or secondary outcomes, suggesting that the intervention strategies achieved much the same benefits irrespective of participants’ level of HL. In other words, health promotion interventions to improve the population’s knowledge of health-related topics can apparently overcome inequalities relating to participants’ baseline HL levels. In one systematic review of health promotion interventions targeting disease self-management conducted in 2010, only eight of the twenty-four trials analysed the modifying effect of different levels of HL, and findings were mixed regarding how much people’s level of HL influenced the effectiveness of such interventions^(45)^.

Studies assessing HL and salt intake found no evidence of any association between the two variables^(23,28)^. As for HL, FL and salt awareness vis-à-vis salt intake, the only variable significantly associated with the salt intake (in one study) was food salt awareness, when it came to the choice of foods. This might seem paradoxical because applying a knowledge of salt content when choosing what food to eat is considered an advanced form of (interactive and critical) FL behaviour. It demands the ability to judge whether a food contributes to healthy nutrition and to distinguish between more healthy and less healthy options^(46)^. This requires a good functional FL, such as knowing the recommendations and being able to read and understand food labels^(46)^. The authors nevertheless conclude that, whatever the potential of HL, FL and specific salt awareness, the high Na content in processed foods may hamper consumers’ efforts to reduce their overall Na intake^(28)^. In fact, previous studies have demonstrated that hedonic response to salt preference depends on a complex interplay of physiological, genetic, psychological and developmental factors and that high salt intake could be the expression of sensory and behavioural factors^(47,48)^. On the other hand, an experimental study^(49)^ found salt taste sensitivity unrelated to the real salt intake, whereas self-reported eating habits were associated with the actual salt intake. This would support the feasibility of limiting individuals’ salt intake effectively through education and training.

The only study based on social cognitive theory hypothesised that self-efficacy and capability (e.g., HL) may interact in influencing preventive health behaviours, such as fat intake, but found no such association. Previous studies had found that overeaters feature a strong liking or preference for fat and a strong attraction to more palatable food, the palatability consequently overriding any satiety signal being triggered by the fat^(50)^. Such a scenario suggests a weak satiety response to fat. It was also demonstrated that high-fat eaters had higher levels of diet-induced thermogenesis and higher leptin levels, suggesting that some individuals stay lean on high-fat diets due to a particular genotype^(51)^. Both these studies suggest that physiological mechanisms could explain dietary fat intake, but also in a complex interplay with psychological aspects. For instance, fat addiction could have important psychological determinants relating to motivation, depression, anxiety and reasoning that need to be carefully examined^(52)^. These considerations and complex relationships should have important implications in how we interpret the influence of HL on dietary behaviour and how we devise intervention to address eating problems and promote healthy lifestyles, especially in adults^(53,54)^.

### Strengths and limitations

While a low HL has been linked to numerous unhealthy behaviours and poor health outcomes, this is the first study (to our knowledge) to systematically review the evidence of the association between people’s HL and their dietary intake of sugar, salt and fat – the three main elements considered in recommendations for a proper diet to prevent non-communicable diseases.

Our systematic review has a few limitations. First, the various studies adopted different tools to measure both HL and dietary intake of the elements considered, making the results difficult to compare and preventing any meta-analysis. The use of a limited number of standardised measuring tools in future studies would facilitate comparisons between international scenarios. Second, some of the samples considered in our review were drawn from particular categories of the population that might be seen as vulnerable (i.e., ethnic minorities) and may not be representative of the general population. The characteristics of the target population could influence the level of association between the variables analysed. Further studies could try to clarify the possible influence of different socio-demographic characteristics, such as age, in mediating the association between HL and health-related behaviour. We also found only one publication describing an intervention programme that enabled us to examine whether HL mediated the efficacy of a health-promoting intervention. Further studies on HL and health-related behaviour would be useful to shed more light on these topics.

## Conclusion

This systematic review concerning the association between HL and the dietary intake of sugar, salt and fat revealed no evidence of any such association for salt and fat, while for sugar, the results are mixed: only about half of the studies considered found an association between a higher level of HL and a more limited sugar consumption. The hypothesised link between health promotion and education strategies and an improvement in HL – with a view to influencing intermediate outcomes such as eating habits – should be tested in further research because this first attempt to summarise the early empirical evidence is inconclusive. More work is also needed to better understand the moderating effects of HL on the outcomes of health promotion interventions.

## Supplementary material

For supplementary material accompanying this paper visit https://doi.org/10.1017/S1368980020002311. click here to view supplementary material
