# Supplementary material for: Association between health literacy and dietary intake of sugar, fat and salt: a systematic review
Source: Public Health Nutr. 2020 Aug 3;24(8):2085–97. doi: 10.1017/S1368980020002311 (PMC8145460; doi:10.1017/S1368980020002311)
Supplement: Supplementary file 1 [file S1368980020002311sup001.docx]

Appendix 1

Search strings - Pubmed:

(health literacy[Title/Abstract] OR literacy[Title/Abstract]) AND (sugar*[Text]) OR (sweet*[Text]) OR (sport drink[Text]) AND "humans"[MeSH Terms] AND English[lang]);

(health literacy[Title/Abstract] OR literacy[Title/Abstract]) AND (salt*[Text]) OR (salty[Text]) OR (sodium[Text]) AND "humans"[MeSH Terms] AND English[lang]);

(health literacy[Title/Abstract] OR literacy[Title/Abstract]) AND (fat[Text]) OR (fats[Text]) OR (fatty[Text]) AND "humans"[MeSH Terms] AND English[lang]);

Search strings - Scopus:

(TITLE-ABS-KEY-AUTH (health AND literacy) AND (TITLE-ABS-KEY-AUTH (sugar*) OR TITLE-ABS-KEY-AUTH (sweet*) OR TITLE-ABS-KEY-AUTH (sport drink))) AND (LIMIT-TO (LANGUAGE, "English"))

(TITLE-ABS-KEY-AUTH (health AND literacy) AND (TITLE-ABS-KEY-AUTH (fat) OR TITLE-ABS-KEY-AUTH (fats) OR TITLE-ABS-KEY-AUTH (fatty))) AND (LIMIT-TO (LANGUAGE, "English"))

(TITLE-ABS-KEY-AUTH(health AND literacy) AND (TITLE-ABS-KEY-AUTH(salt*) OR TITLE-ABS-KEY-AUTH(sodium*) OR TITLE-ABS-KEY-AUTH(salty*))) AND (LIMIT-TO (LANGUAGE, "English"))

Appendix 2 - Attached 1: STROBE criteria ratings. Reviewer A (GG).

|  | **Divaris 2012 Acta Odo0tol Sca0d** | **Gu0tzviller**  **2017**  **J Immigr Mi0or Health** | **Irwa0, A.M**  **2016**  **I0ter0atio0al Jour0al of 0ursi0g Scie0ces** | **Joulaei 2018 Progress i0 0utritio0** | **Luta X  2018**  **0utr Metab Cardiovasc Dis** | **0aghibi Sista0i  2017 Eur J De0t** | **Persoskie 2017 Prev Chro0ic Dis** | **Zoell0er 2011 J Am Diet Assoc** |
| --- | --- | --- | --- | --- | --- | --- | --- | --- |
| 1a | 1 | 1 | 1 | 1 | 1 | 1 | 1 | 1 |
| 1b | 1 | 1 | 1 | 1 | 1 | 1 | 1 | 1 |
| 2 | 1 | 1 | 1 | 1 | 1 | 1 | 1 | 1 |
| 3 | 1 | 1 | 1 | 1 | 1 | 1 | 1 | 1 |
| 4 | 1 | 1 | 1 | 1 | 1 | 1 | 1 | 1 |
| 5 | 1 | 1 | 1 | 1 | 1 | 1 | 1 | 1 |
| 6a |  |  |  |  |  |  |  |  |
|  |  |  |  |  |  |  |  |  |
|  | 1 | 1 | 1 | 1 | 1 | 1 | 1 | 1 |
| 6b |  |  |  |  |  |  |  |  |
|  |  |  |  |  |  |  |  |  |
| 7 | 1 | 1 | 1 | 0 | 1 | 1 | 1 | 1 |
| 8* | 1 | 1 | 1 | 1 | 1 | 1 | 1 | 1 |
| 9 | 0 | 0 | 0 | 0 | 1 | 0 | 1 | 0 |
| 10 | 1 | 0 | 1 | 0 | 1 | 1 | 1 | 1 |
| 11 | 1 | 0 | 1 | 1 | 1 | 1 | 1 | 1 |
| 12a | 1 | 1 | 1 | 0 | 1 | 1 | 1 | 1 |
| 12b | 1 | 0 | 0 | 0 | 1 | 1 | 1 | 1 |
| 12c | 0 | 1 | 0 | 0 | 0 | 1 | 1 | 0 |
| 12d |  |  |  |  |  |  |  |  |
|  |  |  |  |  |  |  |  |  |
|  | 1 | 1 | 1 | 0 | 1 | 1 | 1 | 1 |
| 12e | 0 | 1 | 1 | 0 | 1 | 0 | 1 | 1 |
| 13*a | 1 | 0 | 1 | 0 | 0 | 0 | 0 | 0 |
| 13b | 1 | 0 | 1 | 0 | 0 | 0 | 0 | 0 |
| 13c | 0 | 0 | 0 | 0 | 0 | 0 | 0 | 0 |
| 14*a | 1 | 1 | 1 | 1 | 1 | 1 | 1 | 1 |
| 14b | 0 | 0 | 0 | 0 | 1 | 0 | 0 | 0 |
| 14c |  |  |  |  |  |  |  |  |
| 15* |  |  |  |  |  |  |  |  |
|  |  |  |  |  |  |  |  |  |
|  | 1 | 1 | 1 | 1 | 1 | 1 | 1 | 1 |
| 16a | 1 | 0 | 1 | 1 | 1 | 1 | 1 | 1 |
| 16b | 1 | 1 | 1 | 1 | 1 | 1 | 1 | 1 |
| 16c |  |  |  |  |  |  |  |  |
| 17 | 1 | 1 | 1 | 0 | 1 | 1 | 1 | 1 |
| 18 | 1 | 1 | 1 | 1 | 1 | 1 | 1 | 1 |
| 19 | 1 | 0 | 1 | 0 | 1 | 1 | 1 | 1 |
| 20 | 1 | 1 | 1 | 1 | 1 | 1 | 1 | 1 |
| 21 | 1 | 0 | 0 | 1 | 1 | 1 | 1 | 1 |
| 22 | 1 | 0 | 1 | 1 | 1 | 1 | 1 | 0 |
| **Total score** | 26 | 19 | 25 | 17 | 27 | 25 | 27 | 24 |

Appendix 2 - Attached 2: STROBE criteria ratings. Reviewer B (LM).

|  | **Divaris 2012 Acta Odo0tol Sca0d** | **Gu0tzviller**  **2017**  **J Immigr Mi0or Health** | **Irwa0, A.M**  **2016**  **I0ter0atio0al Jour0al of 0ursi0g Scie0ces** | **Joulaei 2018 Progress i0 0utritio0** | **Luta X  2018**  **0utr Metab Cardiovasc Dis** | **0aghibi Sista0i  2017 Eur J De0t** | **Persoskie 2017 Prev Chro0ic Dis** | **Zoell0er 2011 J Am Diet Assoc** |
| --- | --- | --- | --- | --- | --- | --- | --- | --- |
| 1a | 0 | 0 | 1 | 1 | 0 | 1 | 0 | 1 |
| 1b | 1 | 1 | 1 | 1 | 1 | 1 | 1 | 1 |
| 2 | 1 | 1 | 1 | 1 | 1 | 1 | 1 | 1 |
| 3 | 1 | 0 | 1 | 1 | 1 | 1 | 1 | 1 |
| 4 | 1 | 0 | 1 | 1 | 1 | 1 | 1 | 1 |
| 5 | 0 | 0 | 1 | 1 | 1 | 1 | 1 | 0 |
| 6a |  |  |  |  |  |  |  |  |
|  |  |  |  |  |  |  |  |  |
|  | 1 | 1 | 1 | 1 | 1 | 1 | 1 | 1 |
| 6b |  |  |  |  |  |  |  |  |
|  |  |  |  |  |  |  |  |  |
| 7 | 1 | 1 | 1 | 1 | 1 | 1 | 1 | 1 |
| 8* | 1 | 1 | 1 | 1 | 1 | 1 | 1 | 1 |
| 9 | 1 | 0 | 0 | 0 | 0 | 0 | 0 | 0 |
| 10 | 1 | 1 | 1 | 1 | 1 | 1 | 1 | 1 |
| 11 | 1 | 1 | 1 | 1 | 1 | 1 | 1 | 1 |
| 12a | 1 | 1 | 1 | 1 | 1 | 1 | 1 | 1 |
| 12b | 0 | 0 | 1 | 0 | 0 | 1 | 0 | 0 |
| 12c | 0 | 0 | 0 | 0 | 0 | 0 | 0 | 0 |
| 12d |  |  |  |  |  |  |  |  |
|  |  |  |  |  |  |  |  |  |
|  | 1 | 1 | 1 | 1 | 1 | 1 | 1 | 1 |
| 12e | 1 | 1 | 1 | 1 | 1 | 1 | 1 | 1 |
| 13*a | 1 | 0 | 0 | 0 | 1 | 1 | 1 | 0 |
| 13b | 1 | 0 | 0 | 0 | 1 | 0 | 1 | 0 |
| 13c | 0 | 0 | 0 | 0 | 0 | 0 | 0 | 0 |
| 14*a | 1 | 1 | 1 | 1 | 1 | 1 | 1 | 1 |
| 14b | 0 | 0 | 0 | 0 | 0 | 1 | 0 | 0 |
| 14c |  |  |  |  |  |  |  |  |
| 15* |  |  |  |  |  |  |  |  |
|  |  |  |  |  |  |  |  |  |
|  | 1 | 1 | 1 | 1 | 1 | 1 | 1 | 1 |
| 16a | 1 | 1 | 1 | 1 | 1 | 1 | 1 | 1 |
| 16b | 1 | 1 | 0 | 1 | 1 | 1 | 1 | 1 |
| 16c |  |  |  |  |  |  |  |  |
| 17 | 0 | 0 | 0 | 1 | 0 | 1 | 0 | 0 |
| 18 | 1 | 1 | 1 | 1 | 1 | 1 | 1 | 1 |
| 19 | 1 | 0 | 0 | 0 | 1 | 1 | 1 | 1 |
| 20 | 1 | 0 | 1 | 1 | 0 | 1 | 0 | 1 |
| 21 | 0 | 0 | 0 | 0 | 0 | 0 | 0 | 0 |
| 22 | 0 | 0 | 0 | 0 | 1 | 1 | 0 | 0 |
| **Total score** | 22 | 15 | 20 | 21 | 22 | 26 | 21 | 20 |

Appendix 2 - Attached 3: CONSORT criteria ratings applied to Zoellner et al. 2016.

| **CONSORT items** | Reviewer A (GG) | Reviewer B (LM) |
| --- | --- | --- |
| **Title and abstract** | | |
| 1a Identification as a randomized trial in the title | 1 | 1 |
| 1b Structured summary of trial design, methods, results, and conclusions (for specific guidance see CONSORT for abstracts) | 1 | 1 |
| **Introduction** | | |
| 2a Scientific background and explanation of rationale | 1 | 1 |
| 2b Specific objectives or hypotheses | 1 | 1 |
| **Methods** | | |
| 3a Description of trial design (such as parallel, factorial) including allocation ratio | 1 | 1 |
| 3b Important changes to methods after trial commencement (such as eligibility criteria), with reasons | 0 | 1 |
| 4a Eligibility criteria for participants | 1 | 1 |
| 4b Settings and locations where the data were collected | 1 | 1 |
| 5 The interventions for each group with sufficient details to allow replication, including how and when they were actually administered | 1 | 1 |
| 6a Completely defined pre-specified primary and secondary outcome measures, including how and when they were assessed | 1 | 1 |
| 6b Any changes to trial outcomes after the trial commenced, with reasons | 0 | 1 |
| 7a How sample size was determined | 0 | 1 |
| 7b When applicable, explanation of any interim analyses and stopping guidelines |  |  |
| 8a Method used to generate the random allocation sequence | 1 | 1 |
| 8b Type of randomization; details of any restriction (such as blocking and block size) | 1 | 1 |
| 9 Mechanism used to implement the random allocation sequence (such as sequentially numbered containers), describing any steps taken to conceal the sequence until interventions were assigned | 0 | 0 |
| 10 Who generated the random allocation sequence, who enrolled participants, and who assigned participants to interventions | 0 | 0 |
| 11a If done, who was blinded after assignment to interventions (for example, participants, care providers, those assessing outcomes) and how |  |  |
| 11b If relevant, description of the similarity of interventions | 1 | 0 |
| 12a Statistical methods used to compare groups for primary and secondary outcomes | 1 | 1 |
| 12b Methods for additional analyses, such as subgroup analyses and adjusted analyses | 1 | 1 |
| **Results** | | |
| 13a For each group, the numbers of participants who were randomly assigned, received intended treatment, and were analyzed for the primary outcome | 1 | 1 |
| 13b For each group, losses and exclusions after randomization, together with reasons | 1 | 1 |
| 14a Dates defining the periods of recruitment and follow-up | 1 | 1 |
| 14b Why the trial ended or was stopped | 0 | 0 |
| 15 A table showing baseline demographic and clinical characteristics for each group | 1 | 1 |
| 16 For each group, number of participants (denominator) included in each analysis and whether the analysis was by original assigned groups | 1 | 1 |
| 17a For each primary and secondary outcome, results for each group, and the estimated effect size and its precision (such as 95% confidence interval) | 1 | 1 |
| 17b For binary outcomes, presentation of both absolute and relative effect sizes is recommended |  |  |
| 18 Results of any other analyses performed, including subgroup analyses and adjusted analyses, distinguishing pre-specified from exploratory | 1 | 1 |
| 19 All important harms or unintended effects in each group (for specific guidance see CONSORT for harms) | 0 | 0 |
| **Discussion** | | |
| 20 Trial limitations, addressing sources of potential bias, imprecision, and, if relevant, multiplicity of analyses | 1 | 1 |
| 21 Generalizability (external validity, applicability) of the trial findings | 1 | 0 |
| 22 Interpretation consistent with results, balancing benefits and harms, and considering other relevant evidence | 1 | 1 |
| **Other information** | | |
| 23 Registration number and name of trial registry | 1 | 0 |
| 24 Where the full trial protocol can be accessed, if available | 0 | 0 |
| 25 Sources of funding and other support (such as supply of drugs), role of funders | 1 | 0 |
| **Total CONSORT score** | 26 | 25 |
